# Real-Time PCR as an Alternative Technique for Detection of Dermatophytes in Cattle Herds

**DOI:** 10.3390/ani11061662

**Published:** 2021-06-02

**Authors:** Dominik Łagowski, Sebastian Gnat, Aneta Nowakiewicz, Aleksandra Trościańczyk

**Affiliations:** Department of Veterinary Microbiology, Faculty of Veterinary Medicine, University of Life Sciences in Lublin, Akademicka 12, 20-033 Lublin, Poland; dominik.lagowski@up.lublin.pl (D.Ł.); aneta.nowakiewicz@up.lublin.pl (A.N.); aleksandra.troscianczyk@up.lublin.pl (A.T.)

**Keywords:** dermatophytes, cattle, ringworm, *Trichophyton verrucosum*, *Trichophyton benhamiae*

## Abstract

**Simple Summary:**

Fungal infections of the skin, hair, and nails in humans and animals are the most prevalent mycoses worldwide, with a high economic burden. The high degree of transmissibility poses an epidemiological threat and gives these infections a significant importance as zoonoses. Hence, the control of the presence of dermatophytes in herds of cattle and other species of farm animals should be routinely performed. The ongoing improvements in the field of fungal detection techniques give new scope for clinical implementations in specialized laboratories and hospitals or veterinary clinics, including the monitoring of disease and the detection of side effects of drugs and environmental risks. This study aimed to evaluate the analytical specificity and clinical application of direct sample real-time PCR by comparison with direct microscopy and culture methods. The pan-dermatophyte primers in the qPCR technique facilitated detection of the presence of the genetic material of dermatophytes with greater specificity than the microscopic examination. Moreover, this article describes an interesting case of the isolation of two different species of dermatophytes from the same clinical lesion, i.e., *Trichophyton verrucosum* and *T. benhamiae*.

**Abstract:**

Dermatophytes are filamentous fungi with the ability to digest and grow on keratinized substrates. The ongoing improvements in fungal detection techniques give new scope for clinical implementations in laboratories and veterinary clinics, including the monitoring of the disease and carrier status. The technologically advanced methods for dermatophyte detection include molecular methods based on PCR. In this context, the aim of this study was to carry out tests on the occurrence of dermatophytes in cattle herds using qPCR methods and a comparative analysis with conventional methods. Each sample collected from ringworm cases and from asymptomatic cattle was divided into three parts and subjected to the real-time PCR technique, direct light microscopy analysis, and culture-based methods. The use of the real-time PCR technique with pan-dermatophyte primers detected the presence of dermatophytes in the sample with a 10.84% (45% vs. 34.17%) higher efficiency than direct analysis with light microscopy. Moreover, a dermatophyte culture was obtained from all samples with a positive qPCR result. In conclusion, it seems that this method can be used with success to detect dermatophytes and monitor cowsheds in ringworm cases and carriers in cattle.

## 1. Introduction

Dermatophytes are filamentous fungi with the ability to digest keratinized substrates, i.e., skin, hair, and nails. They are considered the major etiological agents of cutaneous superficial mycoses, often called ringworm [1,2,3,4,5]. These diseases are highly transmissible and clinically varied from mild to severe, depending on the host’s immune status, strain virulence, and other environmental factors [1,6]. The overall prevalence of ringworm in cattle is higher in countries with hotter climates and where the number of animals in the cowshed is high and the contact is closer [3,7,8,9]. Dermatophytosis is commonly considered to be a self-limiting disease, with a course of duration usually varying from 4 to 12 weeks [10]. Appropriate treatment or vaccination may shorten the period with clinical signs [11] and significantly reduce the risk of spread of infection [5].

In most countries, bovine ringworm is considered a relatively harmless disease, with little impact on production parameters and animal welfare [3,11,12]. Control measures are rarely implemented by veterinary authorities or livestock industry [10]. Nonetheless, the use of vaccine became almost routine in 1970–1980 in Hungary [13], Germany [14], Yugoslavia [15], Bulgaria [16], and Norway [17], where approximately 250 million cattle were vaccinated and the results were promising, with a drop in the percentage of infected herds from almost 100% to less than 10% in 1975 and 1% in 1984 [18]. In each of these cases, the vaccination was related to *T. verrucosum* dermatophytosis, although the acquired immunity was extended to trichophytoses with a different etiology [11]. Repeated administration of inactivated or attenuated preparations with a preferable 10 to 14 days interval is recommended to obtain the highest vaccine efficacy [5,11,19,20]. Interestingly, experience from natural outbreaks of ringworm in cattle has proven that the immunity is long-lasting [5,19] and that re-vaccination is not necessary [21].

An interesting issue related to ringworm in cattle is the control of the occurrence of dermatophytes in herds, and also in a carrier state. This issue is especially important in cowsheds where vaccinated and unvaccinated animals are kept. The application of conventional methods of detection and identification of dermatophytes in cattle herds has several drawbacks, as they are laborious and time-consuming and necessitate the experience of a mycological diagnostician [22,23,24]. Technologically advanced methods of dermatophyte identification developed recently include molecular methods based on PCR [25,26]. Moreover, real-time PCR techniques are increasingly frequently used in the diagnosis of human dermatomycoses, due to their high specificity and sensitivity in the detection of dermatophytes directly from clinical samples, and even if cultures assessed with conventional methods are negative [22,25,27]. These qPCR methods have so far only occasionally been used in veterinary mycology. Is it possible to use them for control studies of the presence of dermatophytes in cattle herds?

In this context, the aim of this study was to test the occurrence of dermatophytes in cattle herds using qPCR techniques and compare with the results of conventional methods.

## 2. Materials and Methods

### 2.1. Ethics Standard

This study was carried out in accordance with the relevant national legislation on the use of animals for research. Indeed, this study did not include any experimental procedure that could produce pain, suffering, or distress, in accordance with good veterinary practices. All procedures were performed during routine veterinary and hygienic procedures on the animals.

### 2.2. Dermatophyte Isolates

**Figure 1:**
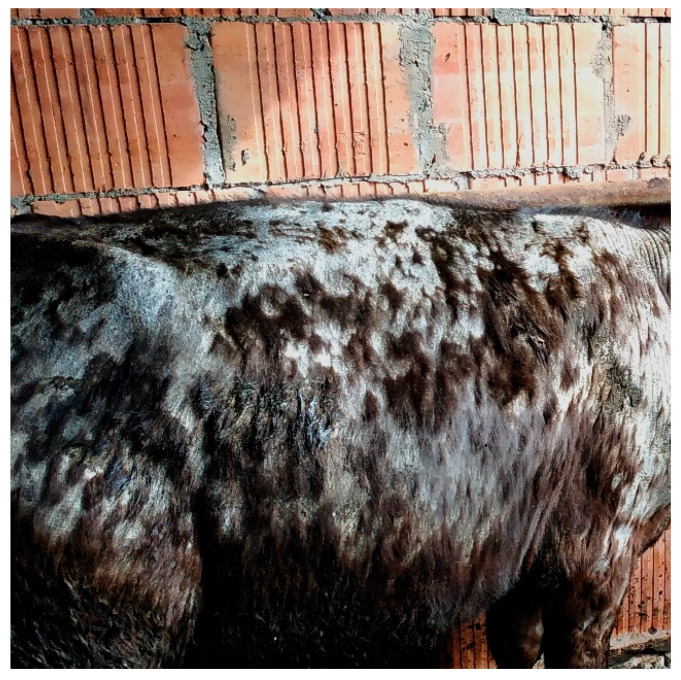
Clinical lesions in the skin and hair of a cow affected by ringworm (camera: Nikon D3300, lens Nikon 18–105 mm VR).

In total, 160 hair samples were collected from asymptomatic cattle (*n* = 120) and animals with ringworm (*n* = 40) from six farms located in different regions of Poland (Table 1). Each farm comprised approximately 700–800 animals aged between 2 weeks and 5 years. The cattle on the farms were kept in a group system, depending on the production purpose. In every cowshed, animals were provided with the standard maintenance, e.g., appropriate nutrition, regular de-worming, and standard vaccination against bacterial and viral infectious disease. None of the animals with symptomatic dermatophytosis had previously been vaccinated against trichophytosis. The clinical signs were similar, i.e., alopecia sites or circa 2 cm excoriations covered with thickened scaling epidermis in multiple locations on the body, primarily on the back, thorax, and neck, with a distinct tendency towards hair loss, pustules, or scabs (Figure 1). From the animals with ringworm, material was collected from the edge of the clinical lesions after cleaning with a cotton swab soaked in 70% ethyl alcohol. Hairs were plucked with sterile forceps to include the root in the samples. The material from the asymptomatic animals was collected using the brush technique. Dermatophytes were isolated in the Department of Veterinary Microbiology, University of Life Sciences in Lublin, Poland. Each sample was divided into three parts to be analyzed with the real-time PCR techniques (1), direct analysis by light microscopy (2), and culture-based methods (3).

### 2.3. Laboratory Diagnostics Procedure

Mycological procedures, i.e., detection and species identification of the isolates, were performed with a few modifications, as described previously by Gnat et al. [23]. For the qPCR technique (P), DNA was extracted using a DNeasy Blood & Tissue Kit (Qiagen, Hilden, Germany), according to the manufacturer’s instructions. A positive result was obtained when the reactions with the pan-dermatophyte primers (Primer F: 5′-AGCGCYCGCCGRAGGA-3′, Primer R: 5′-GATTCACGGAATTCTGCAATTCAC-3′) were positive. The real-time PCR was performed in a final volume of 25 μL containing 12.5 μL 2× of Quanti Tect SYBR green PCR master mix, 0.5 μL (20 pmol) of both forward and reverse primers, 3 μL of DNA, and 8.5 μL of water. The cycling conditions of the reaction performed using Stratagene MX3005P (Agilent Technologies, Santa Clara, CA, USA) were as follows: primary denaturation for 3 min at 96 °C, 45 denaturation cycles of 10 s at 96 °C, annealing for 1 min at 65 °C, and elongation for 30 s at 72 °C. The melting curve analysis was performed in the following conditions: 1 min at 94 °C, 1 min at 65 °C, and 1 min at 94 °C. The qRT-PCR signals were considered positive when the pan-dermatophyte test assay led to a clear rise, resulting in a sigmoid curve and *Ct*-values ≤35. In each run a positive and two negative controls were included. The positive control was DNA extracted from the reference strain *T. verrucosum* CBS365.53 and the negative controls were DNA isolated from healthy hair from a cow and reagents without template DNA. Direct examination of unstained slides from the clinical material in light microscopy (M) was carried out by suspending the samples in a 10% KOH solution with DMSO (dimethyl sulfoxide), which were then viewed at a magnification of 400× (Olympus BX51, Tokio, Japan). Cultures (C) from clinical samples were inoculated onto Sabouraud’s glucose agar (Becton Dickinson, NJ, USA) at 37 °C for 21 days and were identified based on colony texture and production of typical spores. The microscopic analysis of cultures was performed on the 7th, 14th, and 21st day of incubation. Additionally, DNA was isolated from the culture with the phenol-chloroform method [28], and genomic identification was performed by Internal Transcribed Spacer (ITS) amplification and sequencing using the ITS1 (5′-TCCGTAGGTGAACCTGCGG-3′) and ITS4 (5′-TCCTCCGCTTATTGATATGC-3′) primer pair [29]. Amplification was carried out in a T Personal thermal cycler (Biometra GmbH, Goettingen, Germany) with 25 µL of the reaction mixture composed of 12.5 µL Qiagen Taq PCR Master Mix (2.5U Taq DNA Polymerase, 200 pmol of each nucleotide, and 1.5 mmol L^−1^ MgCl_2_) (Qiagen, Hilden, Germany), 10 pmol of each primer (Genomed, Warsaw, Poland), and 1 µL of DNA template. The thermal cycler reaction conditions were as follows: initial cycle at 95 °C for 3 min followed by 30 cycles at 95 °C for 1 min, 50 °C for 1 min, and 72 °C for 1 min, and then an extension cycle of 72 °C for 10 min. Electrophoretic separation of PCR products was carried out in 1% agarose gels. The ITS sequencing reaction was carried out using a BigDye Terminator Cycle Sequencing Kit (Life Technologies, Carlsbad, CA, USA) and the primers ITS1 and ITS4. The PCR mixture (10 µL) contained 2 µL of 2.5× concentrated Ready Reaction Premix, 1 µL of 5× concentrated BigDye Sequencing Buffer, 0.25 µL of the primer at a concentration of 5 pmol (initially 100 pmol), a DNA amplicon at a concentration of 50 ng, and sterile distilled water at a final volume of 10 µL. Two separate reactions were carried out for primers ITS1 and ITS4. PCR was performed in a T Personal cycler (Biometra GmbH) with the following conditions: initial denaturation for 1 min at 96 °C, denaturation for 10 s at 96 °C, annealing of primers for 5 s at 50 °C, and elongation for 4 min 60 °C. The final three stages, i.e., denaturation, annealing of primers, and elongation, were repeated 25 times. The PCR product was purified using an ExTerminator kit (A&A Biotechnology, Gdynia, Poland) and then the DNA sequence was read in a 3500 Genetic Analyzer from Life Technologies (Carlsbad, CA, USA). The ITS nucleotide sequences included in the analyses were compared in pairs and the degree of similarity was determined using ClustalX software (version 1.81), MCD, Dublin, Ireland. Finally, statistical analysis was performed in Statistica ver. 13.1 (StatSoft, Warsaw, Poland). All the mentioned data were statistically analyzed between groups using one-way ANOVA followed by HSD Tukey test. The results were interpreted according to criteria: NS: *p* > 0.05 non-significance, ST: *p* ≤ 0.05.

### 2.4. Enzymatic Activities

The enzymatic activities were evaluated using specific test media, as previously described [27,30]. Briefly, dermatophytes were cultured on Sabouraud’s glucose agar (Becton Dickinson, NJ, USA) and incubated at 37 °C for seven days. Next, an inoculum with a 5 mm diameter from the edge of each culture was transferred onto plates containing the test medium. The following tests were performed: production of keratinase, phospholipase, lipase, protease, urease, elastase, DNase, and gelatinase, and detection of haemolytic activity. Each test was performed in triplicate, and each strain was tested in duplicate in each experiment. The enzymatic activities were expressed as the zone of precipitation surrounding the dermatophyte colonies. A positive result was marked with (+) or (++) depending on the intensity of the expression of enzymatic activity. In the case of detection of haemolytic activity, a transparent zone surrounding the colony indicated complete haemolysis. Particular attention was also paid to the occurrence of double haemolysis around the colony.

## 3. Results

### 3.1. Detection and Identification of Dermatophytes

**Figure 2:**
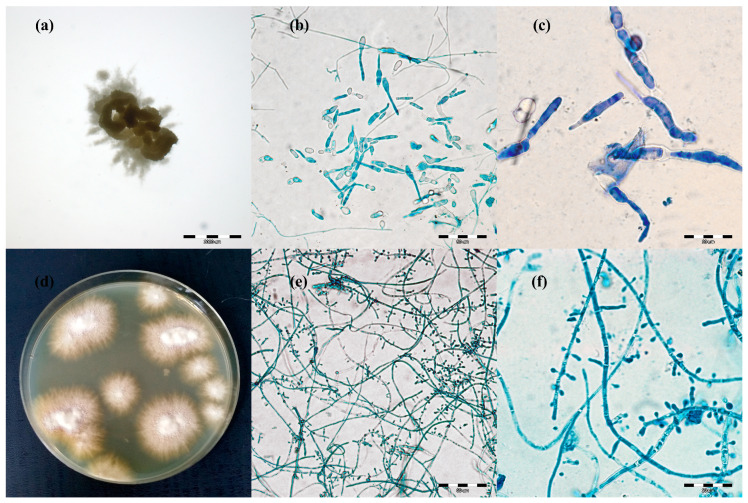
Micro- and macromorphology of two species of dermatophytes isolated from one outbreak of lesions from cattle. Legend: after three passages, two separate pure dermatophyte cultures were obtained: (**a**–**c**), *Trichophyton verrucosum*; (**a**), macromorphology after 21 days of incubation on Sabouraud medium at magnification 4×; (**b**), micromorphology after staining with lactophenol blue at magnification 400×; (**c**), at magnification 1000×; (**d**–**f**), *Trichophyton benhamiae*; (**d**), micromorphology (obverse); (**e**), micromorphology after staining with lactophenol blue at magnification 400×; (**f**), at magnification 1000× (pictures taken with light microscopy Olympus BX51).

The pan-dermatophyte primers in the qPCR technique (P) facilitated detection of the presence of the genetic material of dermatophytes in the sample in 100% of the cases of symptomatic animals (Table 2). Moreover, this method, when used to analyze material from the asymptomatic carrier animals, revealed that 45% of the samples contained the dermatophyte genetic material. In turn, the direct light microscopy analysis of the material (M) sampled from the clinical lesions in the cattle revealed the presence of arthrospores in all samples. In the diagnostics of the material from the asymptomatic animals, elements of the fungus were shown by the direct analysis in 34.17%. In turn, dermatophyte cultures (C) were obtained from 100% and 22.5% of the samples from the ringworm cases and asymptomatic carriage, respectively. This method allowed an identification based on the macro- and micro-morphology of the cultures (Figure 2), followed by confirmation of the results by ITS region sequencing and alignment based on the BLAST algorithm (Basic Local Alignment Search Tool), with sequences deposited in the GenBank database. The analysis of the consistency of the results obtained for the asymptomatic animals revealed that the direct examination by qPCR (P) and the microscopic evaluation (M) showed 75.93% identical results. In 50% and 22.5% of the positive results of qPCR (P) and microscopic examinations (M), respectively, culture (C) was obtained. Additionally, in 25.93% of the samples, positive results were obtained for all three methods tested.

The macro- and micro-morphological image of the 21-day culture demonstrated colonies with a friable texture and a yellow or yellow-orange reverse. The size of the colonies was in the range from 3 to 7 mm. The edges of the colonies were strongly corrugated like a cauliflower surface, and the images of the obverse and the reverse were reminiscent of a star or a turbine rotor (Figure 2a). The micromorphological image on the microscope slides (Figure 2b,c) exhibited sparse, thin, and strongly elongated macroconidia. No chains of circular chlamydospores were observed. *Trichophyton verrucosum* was identified on the basis of morphology.

Different results were obtained for 17 samples. After 7 days of incubation of the material, the macro- and micro-morphological picture was unclear and exhibited not only macroconidia resembling the so-called rat tails, but also numerous small clavate microconidia. Such a morphological picture was not typical of *T. verrucosum* [3,31,32]. After the next 14 days (21st day of culture), the morphological appearance of these samples changed significantly. The size of the colonies was in the range from 8 to 12 mm. On the edge of the colony structure, there was a dense velvety light beige surface, which became suede-like, cauliflower-convex, and brown closer to the center. The micromorphological picture revealed chains of circular chlamydospores, small oval to clavate microconidia laterally inserted at the hyphae, and cigarette-shaped septated macroconidia. The morphological image appeared to be characteristic of more than one species of dermatophytes. Separate cultures were made by grafting a fragment of the mycelia from the margins and from the center of the colonies. After three passages, two separate pure dermatophyte cultures were obtained. One of them was characteristic of *T. verrucosum*, whereas the morphological identification of the other one revealed *T. benhamiae* (Figure 2d–f).

The taxonomic position of all isolates was verified based on a comparative analysis of the ITS sequences of the isolated and reference strains in the GenBank database. The ITS sequences, which were obtained for *T. verrucosum* isolates, in the BLAST exhibited 99% similarity to the *T. verrucosum* CBS365.53, and sequences of 17 isolates morphologically identified as *T. benhamiae* revealed 98–99% similarity to the ITS sequence of *T. benhamiae* CBS 809.72.

### 3.2. Enzymatic Activities

**Figure 3:**
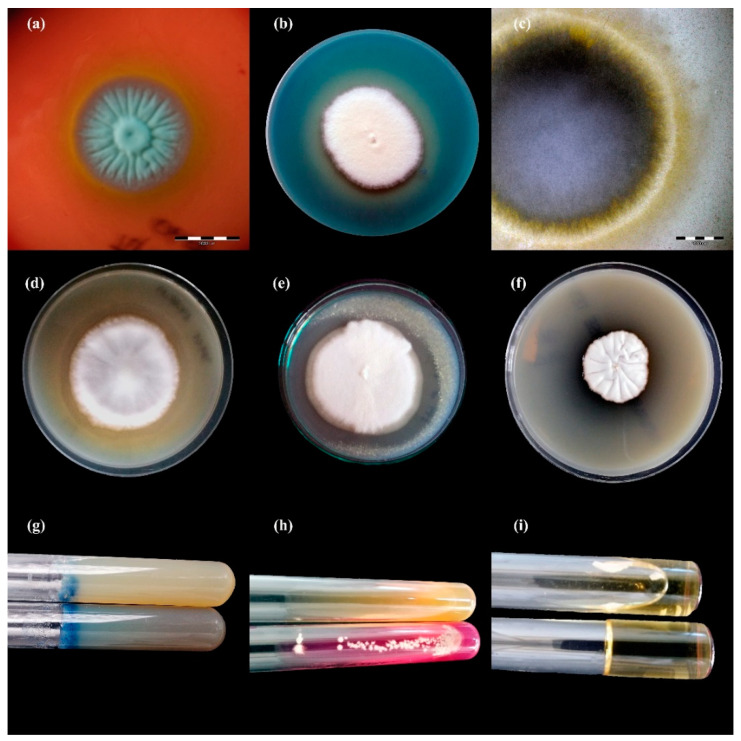
Enzymatic activity of *Trichophyton benhamiae* isolates after 21 days of incubation (picture (**b**,**d**–**i**): Nikon D3300, lens Nikon 18–105 mm VR; picture (**a**,**c**): Olympus SZ61, Tokio, Japan). Legend: (**a**), haemolysis; (**b**), protease; (**c**), lipase; (**d**), phospholipase; (**e**), elastase; (**f**), DNase; (**g**), keratinase; (**h**), urease; (**i**), gelatinase.

**Figure 4:**
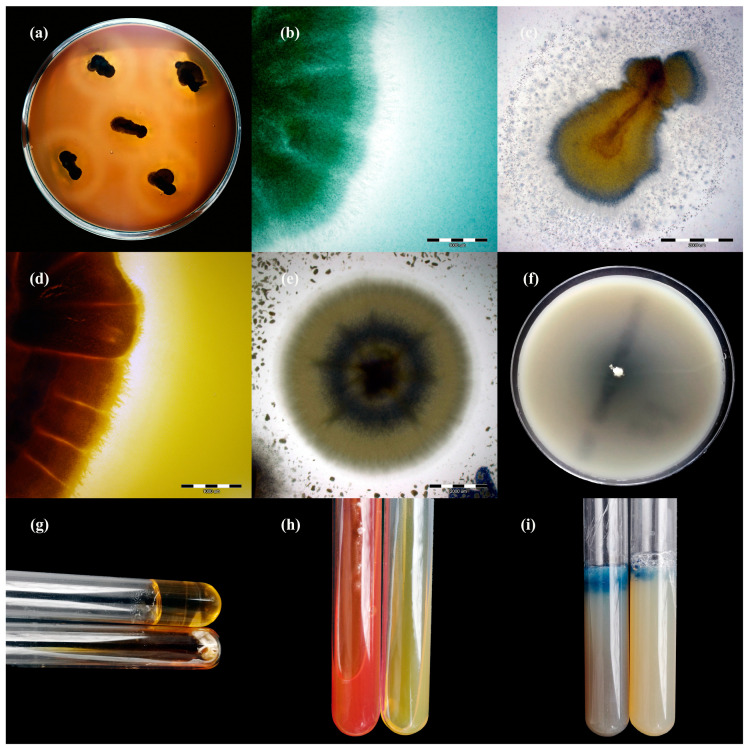
Enzymatic activity of *Trichophyton verrucosum* isolates after 21 days of incubation (picture (**a**,**f**–**i**): Nikon D3300, lens Nikon 18–105 mm VR; picture, (**c**–**e**): Olympus SZ61, Tokio, Japan). Legend: (**a**), haemolysis; (**b**), protease; (**c**), lipase; (**d**), phospholipase; (**e**), elastase; (**f**), DNase; (**g**), gelatinase; (**h**), urease; (**i**), keratinase.

The enzymatic and haemolytic activities of the clinical isolates of dermatophytes were tested in a phenotypic assay (Table 3). In general, the enzymatic activity of the clinical isolates of dermatophytes obtained from cattle was very high. All the clinical isolates of *T. verrucosum* and *T. benhamiae* showed keratinase, phospholipase, lipase, elastase, protease, DNase, urease, and gelatinase activity (Figure 3 and Figure 4). Differences in enzymatic activity were noticeable for lipase, protease, and urease between the *T. verrucosum* and *T. benhamiae* strains. Additionally, the *T. verrucosum* strains produced bizonal haemolysis around the colonies, in contrast to the single and weak haemolysis caused by the *T. benhamiae* isolates. No differences in enzymatic activity were observed between isolates obtained from symptomatic and asymptomatic animals.

## 4. Discussion

Ringworm is considered an important animal disease and has been described in veterinary reports for many years. Movement restrictions on infected herds, hygienic measures, and treatment of infected animals have had little impact on the disease situation worldwide [11,33,34]. The immunoprophylaxis plays an essential role in the control of ringworm on cattle farms, and vaccines are commonly used for both prophylactic and therapeutic purposes [11]. Many factors observed at farm and industry levels justified the introduction of the vaccine on a wide scale in the early 1970s [17], and the cost–benefit considerations supported the importance of its use [11,35]. More than 50 years after the first widespread use of vaccines against trichophytosis, these preparations are still used with an essentially unchanged composition and dosing [36]. Most commercially available vaccines contain live attenuated or inactivated *T. verrucosum* strains, with spores and other fungal elements providing ample exposure of surface antigens. Interestingly, to be suitable for use in production of a vaccine, dermatophyte strains should exhibit good growth and intensive sporulation, especially of only one type of spore [11]. Sarkisov and Nikiforov [18] revealed that microconidia are responsible for immunogenicity; therefore, this fungal element should be contained in the vaccine in the greatest quantity. The effectiveness of vaccination is shown in our study. In the farms in eastern Poland, where vaccinations were performed on a small scale, the highest percentage of symptomatic infections was shown, and all samples taken from the asymptomatic animals were positive. In contrast, other farms with high levels of vaccine use had fewer cattle with infections and lower levels of carriage.

The widespread application and the long time period since the introduction of the vaccine has led to a significant reduction in the prevalence of symptomatic ringworm in cattle [3,36]. Currently, cattle ringworm is noted at certain times of the year, i.e., autumn and spring, and is most often regarded as self-limiting after 3–5 weeks without application of any additional medicinal preparations [37,38]. As a result, veterinarians refrain from performing mycological tests in cattle, even in symptomatic ringworm cases. This leads to a situation where the control of dermatophytes in cattle herds is not supervised and the presence of pathogens in animal hair is underestimated. Perhaps the explanation for this can be found in the time-consuming and labor-intensive mycological procedures, the effectiveness and specificity of which are also low. The solution to this situation may be the use of the qPCR method for detection of dermatophytes in cowsheds, the usefulness of which was assessed in this study.

Our study has proved that the real-time PCR technique with pan-dermatophyte primers detects the presence of dermatophytes in samples with a 10.84% (45% vs. 34.17%) higher efficiency than direct analysis in light microscopy. Moreover, the agreement of the positive qPCR result with the dermatophyte culture was 10.98% (50% vs. 39.02%) higher for this method than for the microscopic examination. Gnat et al. [23] revealed that the qPCR method with pan-dermatophyte primers detected dermatophytes in a sample with a 24.5% (85.4% vs. 60.9%) and 53.7% (85.4% vs. 31.7%) higher efficiency than direct analysis in fluorescence and light microscopy, respectively. This result was therefore much better than that obtained in the present research, the reasons for which can be found in the greater diversity of the analyzed samples. For a homogeneous group of cattle hair samples, this method is slightly less effective. Ohst et al. [22] reported that preselecting microscopically positive samples for real-time PCR analysis increases the proportion of molecularly positive results up to 30%. Nevertheless, this diagnostic approach seems to be incorrect. The lower efficiency of direct microscopic examination than qPCR analysis may lead to omission of potentially positive samples. A certain limitation of qPCR with pan-dermatophyte primers may be the inability to identify the species of dermatophytes. Only the extension of the method with further specific real-time reactions with ITS-oriented primers makes it possible to identify four species- (*Trichophyton rubrum, T. violaceum, T. verrucosum,* *Epidermophyton floccosum*) and five species-complexes of dermatophytes (*Trichophyton interdigitale* complex, *T. schöenleinii* complex, *T. tonsurans* complex, *Microsporum canis* complex, *Arthroderma benhamiae* complex) [22,23]. Nevertheless, the test is then complicated and cost-inefficient and, from the point of view of the detection of dermatophytes in cattle herds, species identification seems to be completely unnecessary.

Interestingly, in our study, the mycological analysis showed the co-existence of two different species of dermatophytes in the same clinical lesion in both symptomatic and asymptomatic cattle infection. One of them was the widely described cattle pathogen, *Trichophyton verrucosum* (member of the *Trichophyton benhamiae* complex) [5,10,32]. Bovine ringworm caused by *T. verrucosum* is endemic in many countries worldwide, with a relatively high frequency of *T. verrucosum* isolation in Greece (1.8%) [7], France (1.53%) [12], Poland (1%) [3], and Slovenia (0.9%) [39], compared to Italy (0.04%) [8], the United States (0.03%) [35], the Czech Republic (0.01%) [40], and Germany (0%) [41]. In Europe, the incidence is correlated with traditional outdoor breeding, which allows free wandering and gathering of animals [3,12]. The second species in the same clinical lesion was the dermatophyte *Trichophyton benhamiae* sensu stricto (member of the *Trichophyton benhamiae* complex), an important zoonotic pathogen with increasing infection rates worldwide over the last 15 years [42,43,44]. This dermatophyte infection has been reported in Switzerland [44,45], Germany [46,47], France [48], Belgium [49], the United Kingdom [50], and Japan [43]. *T. benhamiae* was formerly known as *Trichophyton* species of *Arthroderma benhamiae*; this species was considered part of the *T. mentagrophytes* species complex [4]. Previously, *T. benhamiae* was often misdiagnosed as *Microsporum canis* [51,52] or *T. mentagrophytes* var. *porcellae* [42] due to the similar colony color, or as *T. interdigitale* due to a microscopic similarities [51]. In the literature, it was suggested that guinea pigs and other small rodents are the main source of transmission [51], whereas rabbits [43], dogs [53], or even porcupines [54] are a less common reservoir. Infected companion animals commonly show no or only mild clinical signs, such as alopecia and crusts [51]. So far, only one case of *T. benhamiae* infections in cattle with the co-existence with *T. verrucosum* has been described in the literature, by Łagowski et al. [55]. In their study, the researchers suggest that infection by these two species is competitive. This is somewhat at odds with the carrier state in which these species have also been found. This occurrence is instead complementary, in the specific ecological niche of cattle hair. More extensive research and a more precise differential diagnosis of ringworm in cattle are needed to answer this question.

The enzymatic activity assays of these two species occurring in the same clinical lesion fit well in this regard. A distinct tendency indicating enzymatic activity, which was not species specific, was revealed in our study. Wawrzkiewicz et al. [56] suggested that the keratinolytic activity of dermatophyte strains is connected with the fungal cell, and the enzyme is produced extracellularly only in the case of *T. verrucosum* strains. Thus, keratinolytic activity can be directly linked to the presence of dermatophyte mycelium, and its increase is associated with stronger pathogenicity [57,58]. However, we did not notice such a relationship, and the keratinolytic activity between the isolates of *T. verrucosum* and *T. benhamiae* was comparable. Moreover, literature reports indicate that the induction of keratynolytic activity is connected with a suitable substrate rather than the amount of enzyme protein in the culture [59,60]. Our results indicate that the activity of *T. verrucosum* keratinase is not definitely stronger than in the case of *T. benhamiae;* therefore, the host range cannot be clearly determined. This finding is in contrast with the studies conducted by Mercer et al. [61] and Gnat et al. [59]. The researchers conclude that the accumulation of keratinase does not correlate positively with a higher intensity of natural keratin degradation, and the predisposition of enzymes resulting from the adaptation of the fungus to the natural host may play a key role. This dependence was not noticeable in the present study. This may indicate that clinical isolates of *T. verrucosum* are weakened and have a very low virulence. This effect is probably due to the asymptomatic carrier status in cattle, during which some enzymatic activity is lost or inhibited [30].

Moreover, there has also been some speculation on the possible primary transmission of *T. benhamiae* to cattle. In fact, the reservoirs of these fungi, i.e., *T. verrucosum* and *T. benhamiae,* and the possible routes of infection by these pathogens are completely different [12,62]. The origin of ringworm and the carriage status in cattle induced by *T. benhamiae*, which appeared in a relatively large percentage, is an intriguing issue. As suggested in the scientific literature, guinea pigs, hamsters, and rats may be potential carriers of this zoophilic dermatophyte [44,63]. Given this information, it seems that wild rodents were the most probable causes of the infection in the case described in the present study. Although the farm workers did not notice a single rodent, either in the cowsheds, or during the transport of imported cattle, feces indicating the presence of rodents were noticed during cleaning works in the cowsheds. The spread of fungal arthrospores between cattle that are crowded and have direct contact is very easy. However, no dermatophytes could be isolated from the litter in the cowshed, which was regularly cleaned and disinfected. Therefore, it seems that secondary mechanical transmission between animals is more important in spreading the disease than transferring infectious elements of dermatophytes from litter. In addition, the weakening of the host associated with stress, diet, and poor environmental conditions (heat, humidity, or overcrowding) may have caused the outbreak of the previously dormant pathogen [3,6,64].

## 5. Conclusions

Veterinarians agree that laboratory diagnostics and unambiguous detection of dermatophyte pathogens in cattle herds is essential for control strategies, the isolation of infected animals, and management of epidemic outbreaks. In the field of medical mycology, the dawning of the molecular diagnostics age provided the promise of rapid and reliable detection of these fungi from clinical specimens. Hence, this study aimed to evaluate the analytical specificity and clinical application of direct sample real-time PCR in comparison to the direct microscopy and culture methods. It seems that this method can be used with great success to detect and monitor cowsheds in ringworm cases and dermatophyte carriage in cattle. On the other hand, to identify the species of dermatophyte, a culture should be obtained, which can eliminate errors related to, for example, multiple-species infections.

## Figures and Tables

**Table 1:** Data related to the cattle farms where the study was performed.

Localization on Farms	Statistical Data on Farms (Number of Individuals)				
	Animals	Importing *	Vaccinated (Total)	Symptomatic Ringworm/(%) **	Asymptomatic Carriers ***
south-eastern Poland	760	50	690	9/1.18	8
south-western Poland	810	32	700	3/0.37	12
north-western Poland	700	45	670	9/1.29	4
central Poland	750	38	600	5/0.67	7
eastern Poland	720	65	150	12 (9 ****)/1.67	20 (8 ****)
central-eastern Poland	830	76	754	2/0.24	3

*: animals introduced to the herd of cattle in the last year; **: number of cases among all animals on the farm; ***: data from the present study on the basis of positive qPCR results from 20 tested samples from each farm; ****: number of cases with two different dermatophyte species isolated from one individual from the same clinical lesion.

**Table 2:** Diagnostic effectiveness of tested methods in relation to clinical material taken from symptomatic and asymptomatic dermatophyte infections.

Type of Infection	Method (Number of Positive Animals/% of Positive Results)			Compatible (Number of Animals/% of Consistent Results)			
	qPCR (P)	Microscopy (M)	Cultures (C)	(P)-(M)	(P)-(C)	(M)-(C)	(P)-(M)-(C)
symptomatic	40/100%	40/100%	40/100%	40/100%	40/100%	40/100%	40/100%
asymptomatic	54/45% ^ST^	41/34.17%	27/22.5%	41/75.93%	27/50%	16/39.02%	14/25.93%

^ST^: significantly highest effectiveness of method (assessed by ANOVA followed by HSD Tukey test using the Statistica (Windows v13.1); StatSoft, Warsaw, Poland).

**Table 3:** In vitro enzymatic activity of dermatophyte isolates obtained from cattle after 21 days of incubation.

Isolates	Keratinase	Phospholipase	Lipase	Elastase	Protease	DNase	Urease	Gelatinase	Haemolysis
*T. verrucosum*	+	+	++	+	+	+	+	+	+double
*T. benhamiae*	+	+	+	+	++	+	++	+	+single

## Data Availability

The data presented in this study are available on request from the corresponding author.
